# Measuring Cultural Dimensions: External Validity and Internal Consistency of Hofstede's VSM 2013 Scales

**DOI:** 10.3389/fpsyg.2021.662604

**Published:** 2021-04-06

**Authors:** Philipp Gerlach, Kimmo Eriksson

**Affiliations:** ^1^Psychology School, Faculty of Business & Media, Fresenius University of Applied Sciences, Hamburg, Germany; ^2^School for Education, Culture and Communication, Mälardalen University, Stockholm, Sweden; ^3^Institute for Futures Studies, Stockholm, Sweden

**Keywords:** cultural dimensions, cultural values, Hofstede, indulgence, power distance, individualism, replication, validation

## Abstract

Cross-cultural comparisons often investigate values that are assumed to have long-lasting influence on human conduct and thought. To capture and compare cultural values across cultures, Geert Hofstede's Cultural Dimensions Theory has offered an influential framework. Hofstede also provided a survey instrument, the Values Survey Module (VSM), for measuring cultural values as outlined in his Cultural Dimensions Theory. The VSM has since been subject to a series of revisions. Yet, data on countries have been derived from the original VSM — and not on one of the revised versions of VSM. We tested three scales (indulgence, power distance, and individualism) from the latest version, the VSM 2013, as part of a larger survey across 57 countries. Two main findings emerged. For one thing, country scores based on the VSM 2013 scales correlated only weakly with country scores of the same cultural dimensions obtained in a large previous study. Thus, the validity of the VSM 2013 is in doubt. For another thing, the internal consistency of the VSM 2013 scales was overall poor, indicating that the scales did not reliably measure well-defined constructs. We discuss implications for cross-cultural research.

## Introduction

Geert Hofstede (b. 1928, d. 2020) has been a towering figure in the empirical research of culture. He famously defined culture as “the collective programming of the mind which distinguishes the members of one human group from another” (Hofstede, 1980, p. 25). In this sense, culture is a shared set of beliefs that has a long lasting influence on human conduct and thought — from everyday interactions between neighbors to abstract principles of how society should be organized. At the core of these beliefs are cultural values, such as views on equality, justice, and liberty that are more or less shared among the members of a society.

**Figure 1:**
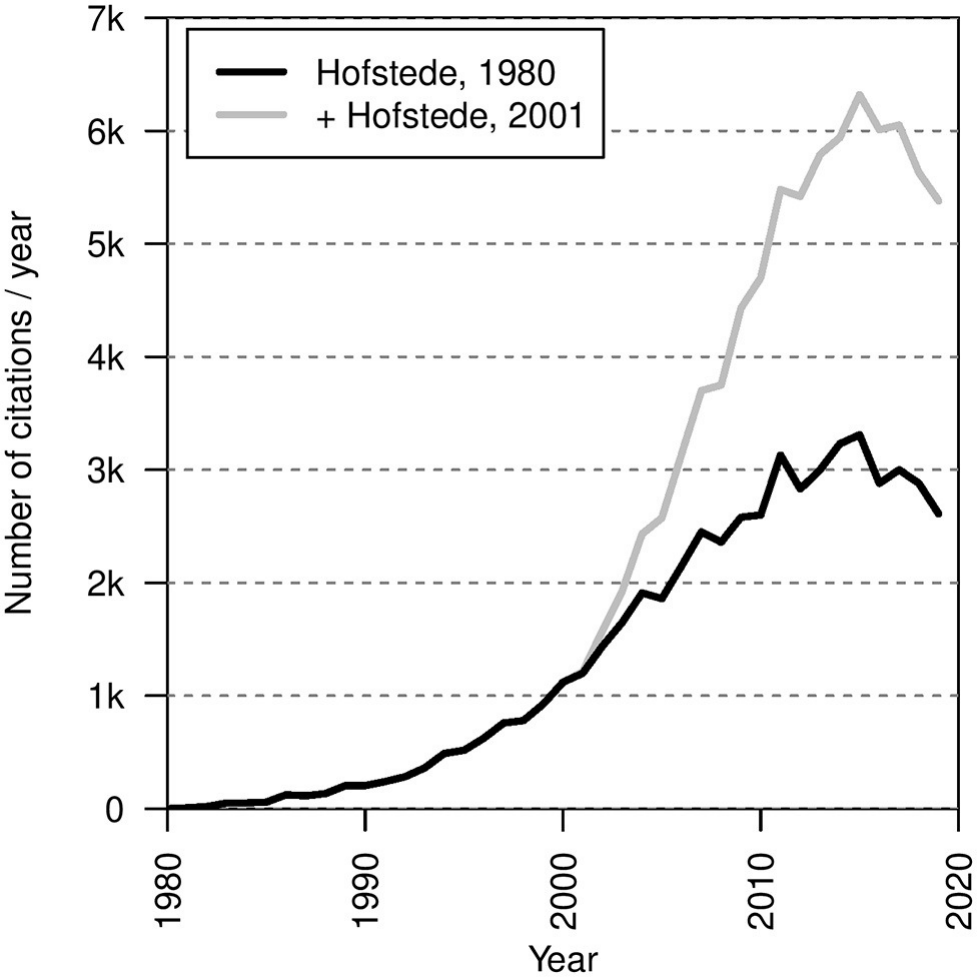
Number of publications in thousands citing Hofstede (1980, 2001) per year (1980–2019). Data taken from a Google Scholar (scholar.google.com) search for all publications. The black line indicates the number of citations of the seminal investigation only (Hofstede, 1980). The gray line includes citations of the second edition (Hofstede, 2001) in addition to the seminal investigation.

To capture and compare cultural values across societies, cross-cultural research has frequently aimed at reducing values to a handful of meaningful dimensions (e.g., Gelfand et al., 2011; Welzel, 2013; Beugelsdijk and Welzel, 2018). Arguably the most influential framework of this type is the Cultural Dimensions Theory of Hofstede himself (Hofstede, 1980, 2001). Since its seminal publication in 1980, Hofstede's Cultural Dimensions Theory has been widely recognized and inspired cross-cultural research across a range of academic disciplines—from sociology to international administration (Orr and Hauser, 2008). Most quantitative measures of culture include at least one dimension that is conceptually similar to those of Hofstede (Taras et al., 2009). Thousands of empirical studies so far have utilized Hofstede's dimensions. As an illustration of its influence, Figure 1 shows how, for decades, Hofstede's initial investigation (and a subsequent revision) has been cited thousands of times every year.

On the one hand, Hofstede's (1980) success is surprising. There are obvious limitations to his work. For example, Hofstede relied on a narrow sample of employees from a single US company (IBM) with mostly highly educated, well-paid, predominantly male, white-collar workers. The study is oversimplifying, ignores within-nation heterogeneity, and assumes a static, single national culture (Baskerville, 2003). It partly rests on faith (Orr and Hauser, 2008). On the other hand, Hofstede's investigation provided a parsimonious approach to study human culture by claiming that various aspects of social conduct and thought are ultimately shaped by just a few cultural dimensions. Arguably, the most attention has been given to two of Hofstede's cultural dimensions: *individualism*, which refers to the extent to which people are primarily expected to look after themselves, and *power distance*, which refers to the degree to which unequal distributions of power are expected and accepted. Hofstede (2011) later added more dimensions, such as *indulgence*, which refers to the extent to which people are allowed to act on their basic human drives. Table 1 provides an overview of all six of Hofstede's cultural dimensions and their common interpretation (Hofstede, 1980, 2001; Minkov and Hofstede, 2010).

**Table 1:** The framework of cultural dimensions and their common interpretation (Hofstede, 1980, 2001; Minkov and Hofstede, 2010).

**Cultural Dimension (vs. opposite pole[s])**	**Common interpretation of the Cultural Dimension (or its high value)**
Individualism (vs. Collectivism)	A preference for a loosely-knit social framework in which individuals are expected to take care of themselves and their immediate families only
Power distance (high vs. low)	The degree to which less powerful members of a society accept and expect that power is distributed unequally.
Masculinity (vs. Femininity)	A preference in society for achievement, heroism, assertiveness, and material rewards for success
Uncertainty avoidance (high vs. low)	The degree to which the members of a society feel uncomfortable with uncertain and ambiguous situations
Long-term orientation (vs. short-term orientation)	The degree to which members of the society are encouraged to thrift and take efforts in modern education as a way to prepare for the future
Indulgence (vs. self-restraint)	The degree to which members of the society are allowed free gratification of basic and natural human drives related to enjoying life and having fun

To measure cultural values as conceived in Cultural Dimensions Theory, Hofstede developed a survey instrument, known as the Values Survey Module (VSM). Its first version, the VSM 80, consisted of items used in Hofstede's original 1980 study (Hofstede, 1980). As described in the manual to the current version, the VSM 2013 (Hofstede and Minkov, 2013), the items have been adapted, replaced, added, and/or removed over the course of a series of revisions of the VSM, numbered 82, 94, 08, and 2013. The many revisions of the VSM could have affected the (country level) internal consistency and validity of the original measurement instrument.

### Controversy Over the Reliability of Cultural Dimensions

At the country-level, Hofstede's seminal 1980 study indicated high internal consistency of the scales used to measure the cultural dimensions (e.g., the three-item power distance scale had α = 0.84 and the six-item individualism scale had α = 0.77). From the introduction of VSM 94 onwards, the scale format was standardized so that each dimension is measured using four items. For the revised scales, Hofstede never provided evidence of internal consistency, instead noting that that the “new items in the new version were chosen because of their similarity to items in reliable other studies, but the reliability of the new dimension scores cannot be proven a priori” (Hofstede and Minkov, 2013, p. 10). Concerns over internal consistency have thus been raised. Spector et al. (2001), for example, used the VSM 94 in a 23-country comparison and found generally poor alpha values. For some scales, alphas were even negative. Hofstede (2002) dismissed their concerns, replying that the validity of VSM country scores was well-established and therefore that the scale must also be reliable. He also suggested that poorly matched samples could have contributed to the poor performance in the study by Spector, Cooper, and Sparks.

As mentioned, Hofstede's (1980) own study was based on relatively homogenous samples of IBM workers. Relying on such homogenous sample is both boon and bane. On the one hand, it serves between country-comparison because potentially confounding variables were relatively constant (e.g., socio-economic background, age, gender). On the other hand, a homogenous sample causes difficulties when generalizing to a heterogeneous population (Orr and Hauser, 2008). To some degree such difficulties can be empirically addressed. For example, Spector and Cooper (2002) controlled for sample demographics in a follow-up investigation and a reply to Hofstede (2002). Yet, they failed to find evidence of improving psychometric performance.

Since the aforementioned debate took place, the VSM has undergone two additional rounds of revisions. These revisions might have improved the psychometric properties of the instrument, potentially making further debate unnecessary. However, to the best of our knowledge the validity of the current version of the instrument, the VSM 2013, has yet to be evaluated. These circumstances motivated our research question: Has the (country level) internal consistency of the VSM 2013 scales improved so that the current VSM provides reliable measures of the cultural dimensions they purport to capture?

### Other Replications of Cultural Dimensions

Empirical studies on Cultural Dimensions Theory include a growing number of replication attempts (for reviews, see Søndergaard, 1994; Kirkman et al., 2006; Orr and Hauser, 2008; Taras et al., 2012). Most replications have compared only a handful of countries and/or only a single cultural dimension). Nonetheless, as replicating data is accumulating, a more thorough picture has been emerging. Most noteworthy, a meta-analysis by Taras et al. (2012) yielded two important findings: first, replications have relatively closely matched the country variation originally observed by Hofstede (1980); second, to a certain extent the match has eroded over time.

An eroding match may have theoretical as well as methodological reasons. For example, the eroding match may be due to the initial findings becoming gradually outdated. That is, national cultures have changed since the 1980s. As a consequence, new theories on cultural values may become necessary. Alternatively, methodological reason may have caused the eroding match. For example, the eroding match may be due to changing measurement instruments. Unlike Spector et al. (2001), many replications relied on other instruments than the VSM (see Taras et al., 2012, p. 331). Moreover, the VSM itself has been subject to many revisions since the first publication. No major replication has been carried out using the latest version, the VSM 2013.

### The Current Study

Our aim in the current study is therefore to conduct a large-scale replication using the VSM 2013. Several different outcomes are conceivable. Case 1: VSM 2013 scores turn out internally consistent and correlate well with the official country-scores provided by Hofstede, which are based mainly on the initial scores published in 1980. Such an outcome would alleviate both theoretical and methodological concerns and suggest that country-based cultural dimensions have been relatively stable over time and can be measured using the latest version of the VSM. Case 2: VSM 2013 scores could lack internal consistency but nonetheless replicate Hofstede's official country-scores. This seems to be the outcome that Hofstede (2002) envisioned in his reply to Spector et al. (2001). It would indicate a fixable methodological concern: items that are not consistent with the main dimension measured by the scale could be removed or replaced. Case 3: VSM 2013 scores could be internally consistent but correlate well only with the most recent country-scores (obtained from the meta-analysis of Taras et al., 2012). Such an outcome would raise theoretical concerns that cultural values are changing over time. Different versions of the VSM may be appropriate to capture these. Case 4: VSM 2013 could be internally *in*consistent and correlate poorly both with Hofstede's initial findings and with the latest country-scores (from Taras et al., 2012). Such an outcome would constitute a failure to validate the VSM 2013 and raise both theoretical and methodological concerns.

In the current study we analyzed data collected in 57 countries from a recent International Study of Metanorms (ISMN; Eriksson et al., 2021). Although the main focus of the ISMN was metanorms (i.e., norms about how to react to norm violations) the survey included several additional measures serving our purposes, namely three scales from the VSM 2013: individualism, power distance, and indulgence. Thus, the dataset provides a unique opportunity to assess the VSM 2013 scales with respect to their internal consistency and their external validity across a wide range of countries. It also complements another recent large-scale cross-cultural study, which failed to replicate other dimensions: masculinity and uncertainty avoidance (Minkov and Kaasa, 2021).

## Methods

### Sample

**Figure 2:**
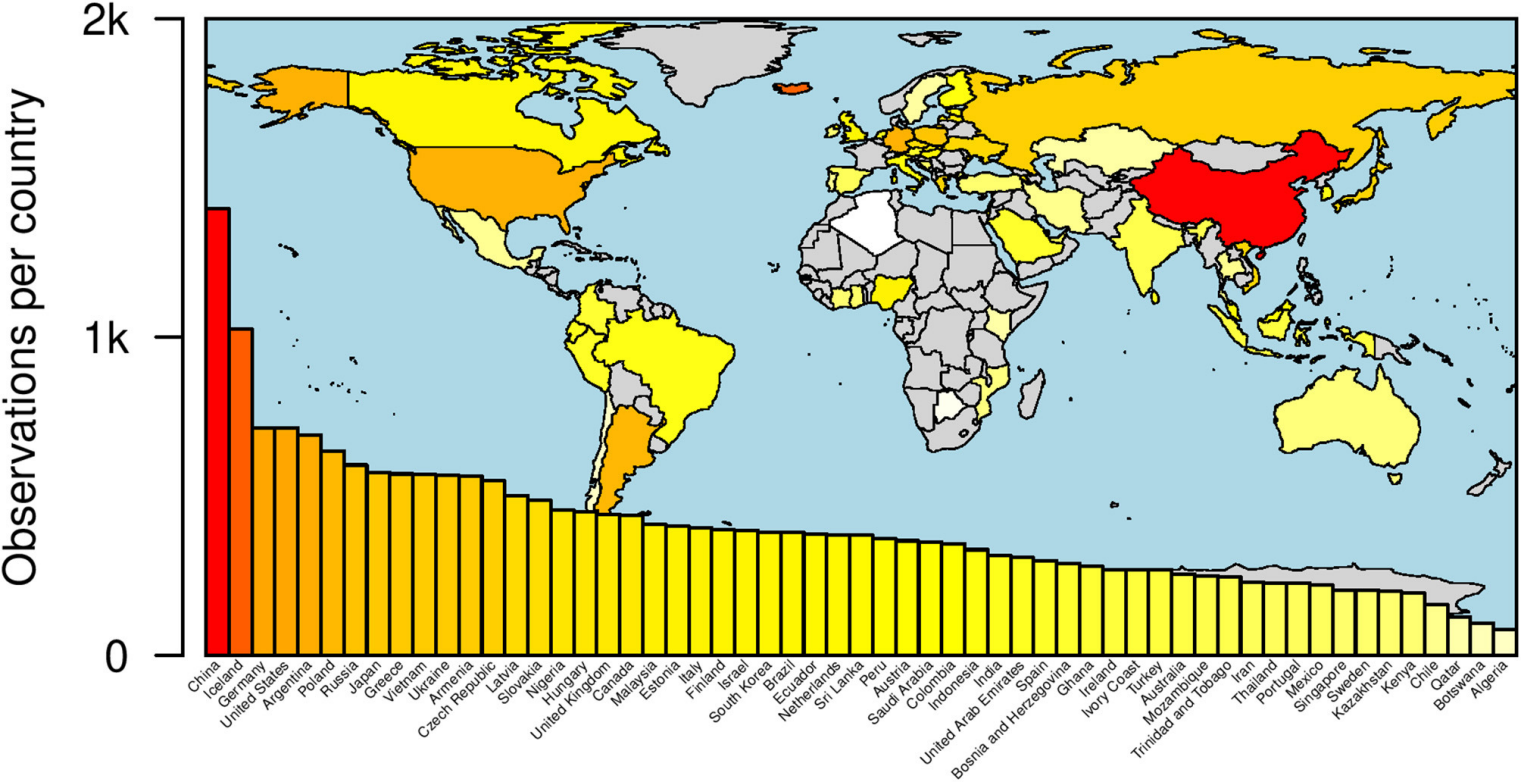
Number of participants in thousands per country included in the ISMN sample. As explained in the Methods section not all countries could be used for all comparisons.

The ISMN data collection is described in detail elsewhere (Eriksson et al., 2021). In brief, local researchers recruited student samples in every participating country, with additional non-student samples recruited in 31 out of 57 countries. Participants completed the survey online or, in a couple of countries, on paper. In almost all countries, the survey was delivered in an official language of the country. The total sample consisted of 22,863 participants (79.1% students, 52.2% women, mean age 24.9 years with a standard deviation of 8.9 years). Sample sizes per country had a mean value of 401.1 and a median of 377. All participating countries provided sample size well above 50, which is the recommended size in the VSM 2013 manual (Hofstede and Minkov, 2013). Figure 2 provides an overview on the participating countries, including their respective sample sizes. Details on sample demographics and survey languages are reported in Supplementary Table 1.

As argued above, for cross-country comparisons, homogenous samples may be preferable (Hofstede, 1980, 2002). This is because factors at the individual level, such as education, gender, and age, could play important roles for the measured cultural values at the collective level (Hofstede, 2001, p. 493). In addition to analyzing the full sample, we therefore analyzed a rather homogenous subsample consisting of 8,620 female students between the age of 18 and 28 years. All 57 countries were represented in this subsample, with a mean sample size of 151.2 and a median of 130.

### Measures of Cultural Dimensions

The VSM 2013 questionnaire provides four items per dimension. The ISMN study covered three cultural dimensions (individualism, power distance, and indulgence) through the following 12 items from the VSM 2013:

Please think of an ideal job, disregarding your present job, if you have one. In choosing an ideal job, how important would it be to you to…

(*01*-R) have sufficient time for your personal or home life;

(*02*-R) have a boss (direct superior) you can respect;

(*04*) have security of employment;

(*06-*R) do work that is interesting;

(*07*) be consulted by your boss in decisions involving your work;

(*09*) have a job respected by your family and friends. In your private life, how important is each of the following to you:

(*11-*R) keeping time free for fun;

(*12*) moderation: having few desires. [1 = of utmost importance 2 = very important 3 = of moderate importance 4 = of little importance 5 = of very little or no importance] × 35

(*16*-R) Are you a happy person?

(*17*) Do other people or circumstances ever prevent you from doing what you really want to? [1 = yes, always 2 = yes, usually 3 = sometimes 4 = no, seldom 5 = no, never] × 40

(*20*) How often, in your experience, are subordinates afraid to contradict their boss (or students their teacher?) [1 = never 2 = seldom 3 = sometimes 4 = usually 5 = always] × 25

(*23*-R) An organization structure in which certain subordinates have two bosses should be avoided at all cost [1 = strongly agree 2 = agree 3 = undecided 4 = disagree 5 = strongly disagree] × 25

In the above list, item numbers refer back to the numbering used in VSM 2013. An “R” indicates that items should be reversely coded. The brackets contain response options and their respective coding. The multiplicative factors are specific item weights (as outlined in the VSM 2013 manual) for three scales: individualism (items no. 01, 04, 06, 09), power distance (02, 07, 20, 23), and indulgence (11, 12, 16, 17). The country score for a scale is calculated as the sum of the country-mean responses to each item of the scale, using item weights and reverse-codings as indicated above^1^.

The Hofstede Insights (2020) website provides country scores on Hofstede's six cultural dimensions. Country scores for individualism and power distance include those obtained in Hofstede's seminal 1980 study as well as scores for other countries obtained in later data collections. Indulgence scores include those published by Minkov and Hofstede (2010). From Hofstede Insights (2020), scores for indulgence were obtained for 48 countries in our study, while scores for power distance and individualism were obtained for 51 countries in our study.

From Taras et al. (2012, Tables 2 and 3, columns 2000s score) we obtained meta-analytic country-scores on power distance for 43 countries in our study and scores on individualism for 47 countries in our study. These meta-analytic country-scores are based on studies that were conducted in the decade 2000–2010, using either VSM or other instruments for measuring Hofstede's cultural dimensions. Note that the dimension of indulgence is more recent and was not included in the meta-analysis of Taras et al. (2012).

## Results

### Internal Consistency of VSM 2013 Scales

Our first research goal was to examine the (country-level) internal consistency of the VSM 2013 scales because reliability is commonly seen as a prerequisite for validity. Following the VSM manual, we calculated country-level Cronbach's alpha values for each cultural dimension. These calculations were carried out both on the full sample and on the more homogenous subsample of young female students. The results are reported in Table 2. Note that, regardless of which sample was used, alpha values were far from the threshold of α > 0.70, commonly used as a rule of thumb for accepting internally consistent scales (e.g., DeVellis, 1991; Nunnally and Bernstein, 1994). We conclude that the items of VSM 2013 do not seem to form internally consistent scales at the country level. In other words, different items do not capture the same cultural variation, suggesting that the VSM 2013 is an unreliable instrument.

**Table 2:** Cronbach's alpha, based on standardized items (at country level), for three scales from VSM 2013.

**Analyses based on**	**Individualism**	**Power distance**	**Indulgence**
Full sample	0.04	−0.71	0.31
Only female college students between 18 and 28 years of age	0.13	−0.34	0.37

*Values of Cronbach's alpha are based on 57 countries for Indulgence and Power distance but only on 56 countries for Individualism, due to one item missing in the questionnaire in Ghana*.

### Validity of Country Scores and Separate Items

To examine the validity of the VSM 2013 scales we used other measures of the same cultural dimensions and correlated them with the scale scores as well as with each separate item. Examining separate items is important as our internal consistency analysis indicated that they do not measure the same construct. When using Pearson correlations with a similar measure to examine validity, prior research has stated that “it is expected that the measure under study correlates strongly (e.g., >0.60 or 0.70) with the similar measure and […] correlations in the range of 0.40–0.60 indicate validity problems or are inconclusive at best” (Post, 2016, p. 1052). Following these recommendations, correlations >0.60 will be considered adequate, correlations <0.40 inadequate, and correlations between 0.40 and 0.60 will be referred to as inconclusive.

Table 3 reports how the VSM 2013 scale scores for individualism and its component items correlated with other measures of individualism. Correlations were often at inadequate levels. Note that correlations with the individualism scores from Hofstede Insights (2020) were consistently inadequate. Individualism scores from Taras et al. (2012) correlated at the inconclusive level with the VSM 2013 individualism scale as well as with two component items: no. 04 (important to have security of employment) and no. 09 (important to have a job respected by your family and friends).

**Table 3:** Pearson correlations between measures in this study and other measures of the cultural dimensions.

**Correlate**	**Scale score**	**Single items (numbered as in VSM 2013)**			
**Individualism**		**Item 1**	**Item 4**	**Item 6**	**Item 9**
Scores from Hofstede Insights (2020) (*n* = 51)	0.23 [−0.07, 0.52]	−0.20	0.20	0.08	0.29
Scores from Taras et al. (2012) (*n* = 47)	0.41 [0.13, 0.63]	−0.20	0.40	0.01	0.45
**Power distance**		**Item 2**	**Item 7**	**Item 20**	**Item 23**
Scores from Hofstede Insights (2020) (*n* = 51)	0.17 [−0.10, 0.39]	−0.10	−0.11	0.14	0.54
Scores from Taras et al. (2012) (*n* = 43)	0.14 [−0.19, 0.42]	−0.02	−0.23	0.17	0.43
**Indulgence**		**Item 11**	**Item 12**	**Item 16**	**Item 17**
Scores from Hofstede Insights (2020) (*n* = 48)	0.28 [−0.01, 0.51]	0.29	0.02	0.40	0.10
Taras et al. (2012) did not provide scores for Indulgence	—	—	—	—	—

*BCa bootstrapped 95% confidence intervals in square brackets. The sample size for the scale and item 4 columns is 1 lower than the reported n value, due to item 4 not being included in Ghana*.

Table 3 also reports how the VSM 2013 scale scores for power distance and its component items correlated with other measures of power distance. Note that the VSM 2013 scale scores for power distance correlated at inadequate levels with the power distance scores from Hofstede Insights (2020) and Taras et al. (2012). A correlation at the inconclusive level was reached for component item no. 23 (“An organization structure in which certain subordinates have two bosses should be avoided at all cost”).

Finally, Table 3 also reports how the VSM 2013 scale scores for indulgence and its component items correlated with indulgence scores from Hofstede Insights (2020). Correlations were generally at inadequate levels, but the inconclusive level was reached for component item no. 16 (“Are you a happy person?”). The meta-analysis by Taras et al. (2012) did not include indulgence. Therefore, we could not correlate our findings with those of Taras et al. (2012).

### Validating the Representativity of Our Samples

Our ISMN country samples were recruited in cities and consisted mostly of young college students. It is therefore legitimate to question if the samples were representative for their national cultures. For this reason, we tested whether the ISMN samples could replicate the country variation in autonomy values^2^ captured by the World Values Survey (WVS), which is a survey on values administered to nationally representative samples. The WVS has been conducted in seven waves since the 1980s, with a new wave roughly every 5 years. For 37 matching countries in our study, data were available from the most recent wave, conducted between 2017 and 2020 (Haerpfer et al., 2020). For another nine matching countries, data were available from an earlier wave accessible (Inglehart et al., 2014). Thus, for a total of 46 countries we could calculate country-scores on autonomy values from the WVS (by averaging the autonomy index measures across all WVS participants in a country, applying the weights provided with the WVS dataset).

Indeed, there were adequate positive correlation between the country means of autonomy values as measured in our samples and in the representative WVS sample, *r* = 0.65 (across *n* = 46 countries; Pearson correlation). This correlation is on par with test-retest correlations for studies using scales from the early VSM versions, which are in the range of *r* = 0.61 to *r* = 0.75 according to Taras et al. (2012, p. 321). We thus conclude that our samples satisfactorily capture the status quo of national cultural variation in values. Hence, we cannot attribute our failure to replicate the variation in Hofstede's cultural dimensions to the lack of representativeness of our ISMN samples.

## Discussion

Prior research has examined the internal consistency of earlier versions of the VSM scales. Hofstede's (1980) own study reported adequate levels of consistency (α > 0.70) for the original version of the VSM scales (Hofstede and Minkov, 2013). Yet, independent tests of the subsequent VSM 94 version suggested that consistency was poor, sparking a debate (Spector et al., 2001; Spector and Cooper, 2002). Here we have contributed to this debate by demonstrating that the methodological problems persist even with the latest VSM scale, the VSM 2013. In brief, our study tested the dimensions individualism, power distance, and indulgence across 57 countries. Our results indicate both poor internal consistency and mostly inadequate validity of the VSM 2013 scales, reflecting another recent failure to replicate the dimensions masculinity and uncertainty avoidance across 47 countries (Minkov and Kaasa, 2021). So why were Hofstede's original country scores fairly well-replicated by the meta-analytic scores of Taras et al. (2012)? We are grateful to a reviewer for pointing out the possibility that primary studies failing to produce scores resembling those from Hofstede (1980) may have been less likely to be published and to make it into the meta-analysis of Taras and colleagues. Although the authors tried to integrate unpublished studies, they did not test to what degree publication bias might be an issue in their data set. Thus, it is possible that publication bias made their replicability estimates artificially high.

We believe that the findings in the current study raise severe methodological concerns for Cultural Dimensions Theory. Inconsistency of a scale implies that, instead of measuring a well-defined construct, it lumps together several relatively independent dimensions. By combining vaguely related items to a single construct, such scale loses precision (Miller, 2002). This is problematic both for interpretation of the measures as well as for the underlying theoretical framework. For instance, prior research has pointed out that Individualism–Collectivism has been defined and operationalized in “overly broad and diffuse ways” (Oyserman et al., 2002, p. 44). However, Hofstede (2002) dismissed concerns about inconsistency as irrelevant on the grounds that the validity of the country scores for the VSM scales was well-established. This is a dubious point, based on faith, and one that should instead be addressed empirically. A meta-analysis (Taras et al., 2012) found that country scores on individualism and power distance obtained in replications correlated at fairly high levels with Hofstede's initial country scores. However, the country scores obtained from the VSM 2013 scales in the current study did not correlate well with either of these previous sets of scores. It seems unlikely that the problem resides with the specific country samples that we used, as the country sample successfully replicated prior measures of autonomy values, another cultural values construct. Instead, we believe that the problem lies with the VSM 2013 instrument itself (cf. Minkov and Kaasa, 2021).

Hofstede's (1980) seminal study was based on a questionnaire that was distributed among IBM employees between the years 1967 and 1972. It consisted of many more items than the ones eventually selected to measure the various cultural dimensions. Hofstede and Minkov (2013, p. 10) freely admit that the “IBM survey questionnaire had not really been composed for the purpose of reflecting international differences in value patterns.” To serve such ends, items were selected *post-hoc*. Orr and Hauser (2008) provide a readable description of the confusing process behind the development of the questionnaire and the *post-hoc* selection of items. The high internal consistency reported for the scales of the original study may in part have been an artifact of this process. As mentioned above, poor internal consistency was observed already in research using the VSM 94 (Spector et al., 2001). Thus, it is likely that the VSM has never been a reliable instrument for measuring cultural dimensions.

Moreover, there are important differences in content between the items included in the current version of the VSM and the original set of items as listed by Orr and Hauser (2008). For instance, of the four VSM 2013 items that comprise the power distance scale, only one (no. 23) is similar to an original item. Note that this item was the one with the strongest correlation to prior measures of power distance. The individualism items similarly have low correspondence to the original items. The scale for indulgence is a special case because the dimension is a late addition to the framework. The country scores on indulgence were based on data from the WVS, using items on happiness, freedom and leisure (Minkov, 2009). Out of the VSM 2013 items, the happiness item (no. 16) had the closest similarity to the WVS items and also exhibited the highest correlation.

### Implications

Cultural values appear to be changing so it is important to update country measures of cultural dimensions (Taras et al., 2012). An important implication of our study is that it is advisable to use other measures than the VSM 2013. For individualism and power distance it may be preferable to stick to other instruments that have been used in prior research, as reviewed by Taras et al. (2012). For indulgence, a better option would be to keep using data from the WVS, which tends to come in a new wave every 5 years.

On a further note, researchers should be careful when using the rather broad generalizations suggested by Hofstede (1980, 2001). The interpretations of the Cultural Dimensions go far beyond what the items actually measure (see Taras et al., 2009). This discrepancy is striking when one compares the actual questionnaire items to the interpretations in Table 1. Weak correlations between separate items yield conceptual insights into the scope of the various Cultural Dimensions. For instance, indulgence item no. 12, on the importance of moderation (having few desires) did not correlate with previous scores on indulgence. Thus, despite the dimension having been described as indulgence vs. restraint, the country variation captured by the WVS data may in fact have little to do with variation in how restraint is valued. Other examples include individualism item no. 1, on the importance of having sufficient time for your personal or home life, or power distance item no. 2, on the importance of having a boss you can respect. These items did not correlate with previous scores on individualism and power distance, respectively, indicating that the values expressed in these items may lie outside the scope of the cultural dimensions.

### Limitations

Our study is limited with respect to comparisons to contemporary replications. Whereas, our dataset was collected in 2019–2020, the meta-analytic scores of Taras et al. (2012) were based on replications from the time period 2000–2010. Moreover, the cultural dimension of indulgence was not included in their meta-analysis and thus could not be tested.

On a further note, the VSM aims primarily at measuring attitudes toward the structures of work organization. Such attitudes can only represent a small subset of what is usually considered cultural values. Yet our samples were, on average, relatively young and thus might have had little work experience so far. Hence, another limitation refers to our samples. The samples were recruited mainly among students in major cities. Although this confers the advantage of easier comparison between countries, much like Hofstede's own sample of IBM employees, it also raises questions about generalizability of findings to, say, rural subcultures. Because cultural variability within countries is potentially large, the use of country averages to compare “national cultures” may simply not live up to the social reality that is intended to be measured.

## Conclusion

In the long run, measuring and comparing national cultural values may be an enterprise that is doomed to fail in an ever globalizing and individualizing world. But in order to draw such broad conclusions we need better measures of culture in the first place. For examples of attempts in this direction, see Yoo et al. (2011) or Beugelsdijk and Welzel (2018).

Cultural dimensions provide a basis for scientific comparisons across cultures. Cultural Dimensions Theory, developed by Hofstede (1980), revolutionized cultural psychology and related fields. In doing so it helped underpinning thousands of decisions of international business practitioners, such as managers, consultants, and cooperate executives. Such practitioners have often turned to Hofstede Insights's (2020) when implementing strategies at different sites. Cultural Dimensions Theory has thus been widely applied, for example to avoid cultural obstacles and to explain failures retrospectively. It is clear that practitioners thereby assumed that cultural dimensions are reliable and valid.

Yet, reliability and validity must be demonstrated empirically. Here we reported an attempt to validate three of Hofstede's cultural dimensions (individualism, power distance, and indulgence) through analysis of data collected in 57 countries using the latest version of Hofstede's survey instrument, the Values Survey Module 2013 (VSM). Our analysis showed that country scores obtained by the VSM were neither internally consistent, nor did they correlate adequately with previously published country scores on these cultural dimensions. This finding raises grave methodological concerns for Cultural Dimensions Theory, making explanations and inferences based on it feeble.

While the introduction of Hofstede's Values Survey Module was an important milestone on the way to study cross-cultural differences in value, a conclusion from our study is that the Values Survey Module does not serve to describe cultural variation in the current world. The time seems ripe for new approaches to study human culture.

## Data Availability Statement

The data analyzed for this study can be found at: https://osf.io/6tgnc/.

## Ethics Statement

Approval of the study protocol was obtained from ethics committees and institutional review boards where required, including: Queen's University (Canada), York University (Canada), Bogotá (Colombia), Institute of Psychology at the Czech Academy of Sciences (Czech Republic), Universidad San Francisco de Quito (Ecuador), United Psychological Research Committee (Hungary), Monk Prayogshala (India), the Trinity College Dublin School of Social Sciences and Philosophy (Ireland), Kwansei Gakuin University (Japan), Aoyama Gakuin University (Japan), United States International University – Africa (Kenya), Sunway University (Malaysia), University of Amsterdam (Netherlands), Komisja ds. Etyki Badań Naukowych Wydziału Psychologii Uniwersytetu SWPS (Poland), Instituto de Ciências Sociais (Portugal), Doha Institute for Graduate Studies (Qatar), Singapore Management University (Singapore), Sungkyunkwan University (South Korea), Universidad de Navarra (Spain), Post Graduate Institute of Medicine (Sri Lanka), Chulalongkorn University (Thailand), American University of Sharjah (United Arab Emirates), University of Kent (United Kingdom), Brunel College of Health and Life Sciences (United Kingdom), University of South Carolina (United States), and New York University (United States). The patients/participants provided their written informed consent to participate in this study.

## Author Contributions

KE conceived of the study, performed the statistical analysis, and wrote methods and results. PG conducted the literature review and wrote the introduction and discussion. Both authors provided critical input and approved the submitted version.

## Conflict of Interest

The authors declare that the research was conducted in the absence of any commercial or financial relationships that could be construed as a potential conflict of interest.

## Footnotes

^1^As discussed in the VSM 2013 manual, scales may be calibrated by addition of an arbitrary scale-specific constant. This is irrelevant for the scale's internal consistency and its rank ordering of countries. We thus calibrated scales to range from 0 and upward, ignoring the constant.

^2^In particular the ISMN samples answered the question “Here is a list of qualities that children can be encouraged to learn at home. Which, if any, do you consider to be especially important?”. The list of alternatives includes, among others, independence, determination/perseverance, religious faith, and obedience. We follow the WVS in calculating an autonomy index, ranging from −2 to +2, by the formula Autonomy = Independence + Determination – Religious faith – Obedience, where each term is 1 if selected by the participant, 0 otherwise. We use the country-mean of the autonomy index as a measure of the autonomy values in the country.
