# Supplementary material for: Measuring Cultural Dimensions: External Validity and Internal Consistency of Hofstede's VSM 2013 Scales
Source: Front Psychol. 2021 Apr 6;12:662604. doi: 10.3389/fpsyg.2021.662604 (PMC8056018; doi:10.3389/fpsyg.2021.662604)
Supplement: Supplementary file 1 [file Table_1.DOCX]

**Supplementary Table 1.** Description of sample characteristics of country, data collection site(s), language of survey, student sample (size, age, gender), and non-student sample (size, age, gender).

|  | | | Student sample | | | Non-student sample | | |
| --- | --- | --- | --- | --- | --- | --- | --- | --- |
| Country | Survey language | City | Size | Mean age | % Female | Size | Mean age | % Female |
| Algeria | Arabic | Oran | 82 | 23.2 | 78.6 |  |  |  |
| Argentina | Spanish, South American* | Córdoba | 214 | 21.8 | 69.6 | 478 | 29.5 | 66.0 |
| Armenia | Armenian | Yerevan | 406 | 21.8 | 67.4 | 157 | 29.1 | 62.5 |
| Australia | English | Melbourne | 255 | 19.6 | 75.5 |  |  |  |
| Austria | German | Linz | 360 | 22.0 | 66.2 |  |  |  |
| Bosnia and Herzegovina | Bosnian-Croatian-Serbian | Banja Luka | 289 | 21.2 | 49.8 |  |  |  |
| Botswana | English | Gaborone | 102 | 20.9 | 82.2 |  |  |  |
| Brazil | Brazilian Portuguese | Saõ Paolo | 262 | 27.8 | 51.0 | 124 | 39.9 | 61.1 |
| Canada | English | Kingston | 210 | 19.8 | 55.6 |  |  |  |
|  |  | Toronto | 231 | 20.8 | 77.5 |  |  |  |
| Chile | Spanish, South American* | Santiago | 159 | 20.9 | 42.3 |  |  |  |
| China | Chinese simplified | Beijing | 289 | 19.9 | 81.1 | 138 | 30.2 | 42.4 |
|  |  | Guangzhou | 233 | 18.9 | 71.0 | 121 | 37.2 | 57.8 |
|  |  | Shenzhen | 622 | 20.2 | 67.2 |  |  |  |
| Colombia | Spanish, South American* | Bogotá | 231 | 20.3 | 53.9 | 119 | 36.5 | 73.5 |
| Czech Republic | Czech | Brno | 360 | 25.6 | 75.6 | 188 | 34.3 | 77.5 |
| Ecuador | Spanish, South American* | Quito | 328 | 21.5 | 62.2 | 53 | 28.8 | 59.1 |
| Estonia | Estonian | Tallinn | 287 | 28.1 | 75.7 | 120 | 32.3 | 85.0 |
| Finland | Finnish | Helsinki | 356 | 28.7 | 76.0 | 38 | 49.8 | 72.0 |
| Germany | German | Cologne | 204 | 23.0 | 60.1 | 98 | 31.6 | 47.9 |
|  |  | Hagen |  |  |  | 413** | 31.5 | 77,5 |
| Ghana | English | Accra | 220 | 20.8 | 62.1 | 60 | 31.3 | 40.0 |
| Greece | Greece | Athens | 160 | 26.1 | 55.3 | 28 | 37.1 | 52.9 |
|  |  | Patras | 231 | 21.6 | 78.7 | 151 | 38.2 | 66.9 |
| Hungary | Hungarian | Budapest | 356 | 21.6 | 76.4 | 96 | 34.6 | 89.6 |
| Iceland | Icelandic | Reykjavik | 834 | 27.0 | 74.3 | 190 | 44.2 | 76.9 |
| India | English | Mumbai | 283 | 18.9 | 87.8 | 31 | 37.0 | 38.5 |
| Indonesia | Bahasa Indonesia | Surabaya | 212 | 19.7 | 86.6 | 120 | 27.6 | 71.1 |
| Iran | Farsi | Teheran | 230 | 21.5 | 71.6 |  |  |  |
| Ireland | English | Dublin | 247 | 20.9 | 57.1 | 23 | 43.6 | 69.2 |
| Israel | Hebrew | Tel Aviv | 265 | 26.6 | 68.7 | 127 | 30.3 | 41.3 |
| Italy | Italian | Rome | 189 | 22.3 | 43.1 |  |  |  |
|  |  | Turin | 212 | 23.4 | 70.0 |  |  |  |
| Ivory Coast | French | Abidjan | 270 | 26.2 | 42.4 |  |  |  |
| Japan | Japanese | Kanagawa | 265 | 20.1 | 42.9 |  |  |  |
|  |  | Osaka-Kobe | 309 | 19.7 | 60.7 |  |  |  |
| Kazakhstan | Russian | Almaty | 201 | 19.7 | 58.2 |  |  |  |
| Kenya | English | Nairobi | 196 | 22.0 | 48.4 |  |  |  |
| Latvia | Latvian | Daugavpils | 436 | 28.7 | 71.8 | 66 | 38.4 | 58.3 |
| Malaysia | English | Kuala Lumpur | 290 | 21.5 | 48.8 | 122 | 33.4 | 58.1 |
| Mexico | Spanish. South American* | Mexico City | 222 | 25.2 | 68.1 |  |  |  |
| Mozambique | Portuguese | Maputo | 250 | 23.1 | 34.3 |  |  |  |
| Netherlands | English | Amsterdam | 379 | 21.7 | 54.8 |  |  |  |
| Nigeria | English | Nsukka | 240 | 22.7 | 58.2 | 217 | 36.1 | 54.2 |
| Peru | Spanish. South American* | Lima | 126 | 29.3 | 68.6 | 240 | 36.8 | 66.9 |
| Poland | Polish | Warsaw | 283 | 27.0 | 83.2 | 359 | 41.8 | 60.4 |
| Portugal | Portuguese | Lisbon | 227 | 24.3 | 88.7 |  |  |  |
| Qatar | Arabic | Doha | 62 | 28.4 | 85.2 | 59 | 29.1 | 89.2 |
| Russia | Russian | Moscow | 382 | 23.0 | 76.5 |  |  |  |
|  |  | St Petersburg | 216 | 21.9 | 73.6 |  |  |  |
| Saudi Arabia | Arabic | Riyadh | 261 | 21.5 | 11.4 | 95 | 32.8 | 58.4 |
| Singapore | English | Singapore | 205 | 22.0 | 68.3 |  |  |  |
| Slovakia | Slovak | Bratislava | 363 | 25.6 | 61.9 | 124 | 39.0 | 43.8 |
| South Korea | Korean | Seoul | 252 | 20.8 | 52.7 | 135 | 39.5 | 55.2 |
| Spain | Spanish | Madrid | 134 | 19.5 | 50.4 | 164 | 42.3 | 35.0 |
| Sri Lanka | English | Colombo | 377 | 23.2 | 60.6 |  |  |  |
| Sweden | Swedish | Linköping | 165 | 24.7 | 53.9 |  |  |  |
|  |  | Stockholm | 40 | 37.4 | 50.0 |  |  |  |
| Thailand | Thai | Bangkok | 228 | 19.8 | 67.9 |  |  |  |
| Trinidad and Tobago | English | Port of Spain | 173 | 24.7 | 74.4 | 73** | 29.0 | 85.7 |
| Turkey | Turkish | Istanbul | 269 | 21.6 | 83.4 |  |  |  |
| United Arab Emirates | English | Sharjah | 307 | 20.0 | 63.8 |  |  |  |
| Ukraine | Ukrainian, Russian | Kiev | 447 | 22.9 | 70.7 | 118 | 38.5 | 55.4 |
| United Kingdom | English | Canterbury | 193 | 19.7 | 80.2 |  |  |  |
|  |  | London | 122 | 19.1 | 89.1 | 127 | 36.4 | 85.4 |
| United States | English | Columbia. SC | 497 | 19.4 | 76.8 |  |  |  |
|  |  | New York | 217 | 21.9 | 74.3 |  |  |  |
| Vietnam | Vietnamese | Hanoi | 568 | 18.7 | 75.8 |  |  |  |
| Total: 57 | 30 | 68 | 18091 |  |  | 4772 |  |  |

| * With a few local lexical changes.  ** Part-time students. |
| --- |
